# Anti-Th17 and anti-Th2 responses effects of hydro-ethanolic extracts of *Aframomum melegueta*, *Khaya senegalensis and Xylopia aethiopica* in hyperreactive onchocerciasis individuals’ peripheral blood mononuclear cells

**DOI:** 10.1371/journal.pntd.0010341

**Published:** 2022-04-25

**Authors:** Gnatoulma Katawa, Essoham Ataba, Manuel Ritter, Oukoe Marthe Amessoudji, Essimanam Rosalie Awesso, Pélagie Edlom Tchadié, Fagdéba David Bara, Fekandine Victoire Douti, Kathrin Arndts, Tchadjobo Tchacondo, Komlan Batawila, Yaovi Ameyapoh, Achim Hoerauf, Simplice D. Karou, Laura E. Layland

**Affiliations:** 1 Unité de Recherche en Immunologie et Immunomodulation (UR2IM)/Laboratoire de Microbiologie et de Contrôle de Qualité des Denrées Alimentaires (LAMICODA)/Ecole Supérieure des Techniques Biologiques et Alimentaires (ESTBA), Université de Lomé, Lomé, Togo; 2 Institute for Medical Microbiology, Immunology and Parasitology (IMMIP), University Hospital Bonn (UKB), Bonn, Germany; 3 Laboratoire de Biologie et Écologie Végétale, Faculté des Sciences (FDS), Université de Lomé, Lomé, Togo; 4 German Center for Infection Research (DZIF), partner site Bonn-Cologne, Bonn, Germany; 5 German-West African Centre for Global Health and Pandemic Prevention (G-WAC), Partner Site Bonn, Bonn, Germany; Istituto Superiore di Sanità, ITALY

## Abstract

Hyperreactive onchocerciasis (HO) is characterized by a severe skin inflammation with elevated Th17-Th2 combined responses. We previously demonstrated the anthelminthic activity of *Aframomum melegueta* (AM), *Xylopia aethiopica* (XA) and *Khaya senegalensis* (KS) used by traditional healers to treat helminthiasis in the endemic area of Togo. However, their effect on severe onchocerciasis is poorly investigated. The present study aimed to investigate the anti-Th17 and anti-Th2 effects of hydro-ethanolic extracts of AM, XA and KS during HO. *Onchocerca volvulus*-infected individuals were recruited in the Central region of Togo in 2018. Isolated peripheral blood mononuclear cells (PBMCs) from both generalized onchocerciasis (GEO) and HO forms were activated with anti-CD3 and anti-CD28 monoclonal antibodies in the presence or absence of the hydro-ethanolic extracts of AM, XA and KS as well as their delipidated, deproteinized and deglycosylated fractions. After 72 hours, cytokines were assayed from cell culture supernatants. Then, flow cytometry was used to investigate the effects of the extracts on cell activation, proliferation, intracellular cytokines and T cells transcription factors. The production of both Th17 and Th2 cytokines IL-17A and IL-5 were significantly inhibited upon T-cell receptor (TCR) activation in the presence of the hydro-ethanolic extracts of AM, XA and KS in HO individuals’ PBMCs *in vitro*. AM and XA inhibited CD4^+^RORC2^+^IL-17A^+^ and CD4^+^GATA3^+^IL-4^+^ cell populations induction. This inhibition was not Th1 nor Treg-dependent since both IFN-γ and IL-10 were also inhibited by the extracts. AM and XA did not interfere with T cell activation and proliferation for their inhibitory pathways. Lipid and protein compounds from AM and XA were associated with the inhibition of IL-17A. This study showed that in addition to their anthelminthic effects, hydro-ethanolic extracts of A*framomum melegueta*, *Xylopia aethiop*ica and *Khaya senegalensis* could downregulate both Th17 and Th2 responses and prevent the severe skin disorder observed.

## Author summary

Severe form of human onchocerciasis also called hyperreactive onchocerciasis (HO) is characterized by skin disorders such as dermatitis. Our previous study showed that concomitant Th2 and Th17 responses play a role in the pathophysiology of the disease. In *Onchocerca volvulus* endemic areas such as Togo, traditional healers (TH) used to treat this disease with medicinal plants such as *Aframomum melegueta* (AM), *Xylopia aethiopica* (XA) and *Khaya senegalensis* (KS). Mass drug administration including ivermectin and albendazole has been used over the past decade to control and eradicate helminths infections. Despite its successes, resistance to ivermectin has been reported and therefore, alternative drugs are urgently needed. We previously confirmed *in vitro* the anthelminthic effects of *Aframomum melegueta* (AM), *Xylopia aethiopica* (XA) and *Khaya senegalensis* (KS). However, their effect on severe onchocerciasis has not been demonstrated. Here, the anti-Th17 and anti-Th2 effects of hydro-ethanolic extracts of AM, XA and KS during HO were investigated. The data showed that in addition to their helminth-killing effects, plant-derived molecules AM, XA and KS downregulated Th2 and Th17 profiles and therefore, could be candidates for the development of new drugs not only for the treatment of helminth-induced inflammatory pathologies but also auto-immune Th2/Th17-dependent inflammatory diseases in general.

## Introduction

Commonly known as river blindness, onchocerciasis is a filarial infection of the eyes and skin transmitted by the bite of black flies (*Simulium spp*) infected with *Onchocerca volvulus* [1]. *Onchocerca volvulus* is one of many parasitic helminthiasis species affecting nearly 17.7 million people mostly in Africa [2]. Onchocerciasis is one of the parasitic helminthiases causing a serious public health problem in tropical regions [3]. The pathogenesis of the disease is linked to the death of the parasite and is characterized by an inflammation leading to skin damage and blindness [4]. The clinical manifestations of this inflammation are intense and bothersome including itching, acute and chronic papular dermatitis, lichenified onchodermatitis, depigmentation (leopard skin) loss of elasticity and structure of the skin, leading to signs of premature skin aging [5]. During clinical manifestations, two polar forms can be distinguished: the asymptomatic generalized onchocerciasis (GEO) or the hyperreactive form (HO), also called sowda presenting a severe skin inflammation which can occur in immunologically hyperactive individuals with chronic papular dermatitis and hyperpigmentation [6]. These individuals are characterized by a low number of parasites (<10 microfilariae (MF) per mg of skin or negative) and an enhanced Th2 immunity [6]. In addition to Th2 cells, we showed that Th17 responses that have been shown to initiate and drive inflammation were also associated with hyperreactive forms [4].

The current drugs used to treat filariasis and onchocerciasis include, doxycycline, ivermectin and albendazole, which are used primarily in combination to shrink MF in the skin [3]. Despite the many successes of helminth control programs, countries in Sub-Saharan Africa and India still face the challenges of transferring these programs into integrated strategies for the control of Neglected Tropical Diseases (NTDs) [7]. Around 99% of the global onchocerciasis burden exist in Africa [8] and thus, there is a need to develop alternative drugs [3] especially since several reports of suboptimal responses to ivermectin have been published [9–12]. These situations may be explained by a development of resistance to ivermectin [13]. Therefore, alternative drugs are urgently needed and traditional medicinal plants are receiving special attention in global health debates. This is visible in the increased investment in traditional herbal medicine research by governments, international agencies and companies [14].

Eighty percent of African people use a form of traditional herbal medicine [15,16]. Traditional medicine is not only used for primary health care needs, but also, it is used as a source of solution to chronic diseases that are constantly growing in the world according to the World Health Organization (WHO) [17]. It is noted that more than 50% of modern medicine drugs are derived from plants [18]. Also, traditional herbal medicine has been bequeathed to the forefront in the strategy of easing and treating severe acute respiratory syndrome (SARS) in China [19]. In Democratic Republic Congo, 55% of people used plants as medicine to treat onchocerciasis [20]. Thus, plants are an important source of molecules that have anti-parasitic, hepatoprotective, anti-viral, anti-inflammatory and immunomodulatory properties [21]. Interestingly, distinct plants like *Aframomum melegueta* (AM), *Xylopia aethiopica* (XA) and *Khaya senegalensis* (KS) are regularly used by traditional healers to treat helminthiases in endemic areas of Togo and it has been shown that these plants have anthelminthic effects *in vitro* [22]. However, the anti-inflammatory activity of these plants remains unknown but need to be explored, since severe inflammation in HO patients are characterized by elevated Th17 and Th2 responses and low or absent parasite burden [4]. Therefore, the present study aimed to investigate the effect of the hydro-ethanolic extracts of *Aframomum melegueta* (AM), *Xylopia aethiopica* (XA) and *Khaya senegalensis* (KS) on elevated Th17 and Th2 responses during HO *in vitro*. The findings highlight initial evidence that plant-derived molecules could downregulate Th2 and Th17 responses and therefore could be used for the treatment of hyperreactive onchocerciasis or helminth-induced inflammatory pathology in particular and auto-immune Th2/Th17-dependent inflammatory diseases in general.

## Methods

Ethics statementThis study was reviewed and ethically approved by the ‘Comité de Bioéthique pour la Recherche en Santé (CBRS)’ of the Health Ministry of Togo (N°043/2016/MSPS/CAB/SG/DPLET/CBRS). Written informed consent was obtained from all participants.

### Study population

**Fig 1:**
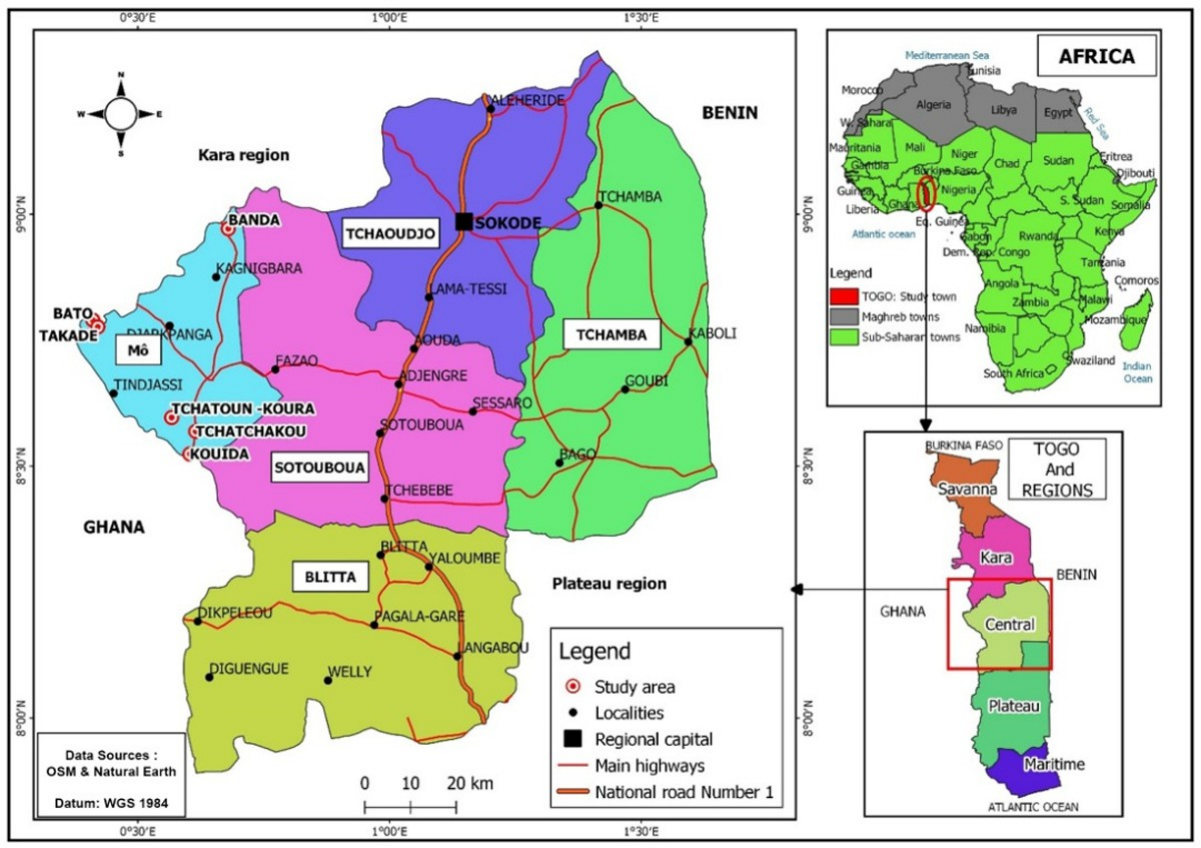
Map of Togo showing the six villages (study area) of the “Préfecture de Mô” in the Central region. **Maps were adapted from:** https://www.naturalearthdata.com/http//www.naturalearthdata.com/download/10m/cultural/ne_10m_admin_0_countries.zip and https://www.naturalearthdata.com/http//www.naturalearthdata.com/download/10m/cultural/ne_10m_admin_1_states_provinces.zip. To adapt maps QGIS 3.20.0 software was used.

In 2018, *Onchocerca volvulus*-infected individuals including both generalized onchocerciasis (GEO) and hyperreactive onchocerciasis (HO) forms were enrolled. Samples were obtained from individuals living in an *Onchocerca volvulus* endemic six (6) villages (Bato, Takadè, Kouida, Tchetchekou, Tchatoukoura and Banda) of the “Préfecture de Mô” in the Central region of Togo (Fig 1). The sample size (n = 237) was calculated using Schwartz formula n = Z^2^ P (1-P) / d^2^ where Z, the accepted risk error is 1.96; d, the precision, is 0.05; P, the prevalence. In the northern and central regions of Togo, the prevalence of onchocerciasis is estimated at 5.7% according to a study conducted by Komlan et al., 2018 [23]. So, using these factors and the prevalence of 5.7%, the minimum sample size is 41. Recruited individuals (237) included adult men and women aged between 18–55 years old. For comparison, samples were collected from *O*. *volvulus* infection free volunteers from the same area and are thus referred here to as endemic normal (EN). All participants with HIV, viral hepatitis and metabolic disorders were not enrolled.

### Parasitological assessment

All individuals received clinical examination to assess the presence of nodules and/or skin lesions and skin snips were obtained to detect dermal MF as previously described [24–26]. In short, two skin snips were collected from the left and right iliac crest using a corneo-scleral (Holth) punch (Koch, Hamburg, Germany), and incubated in 100μl NaCl (0.9%) overnight at room temperature in 96 well microtiter plates. Thereafter, MF were diagnosed and counted using an optical microscope. Other helminths’ infections (schistosomes, ascaris and hookworms) were diagnosed using standard methods (Kato-Katz and urine analysis), but no other helminth infections could be detected in all individuals.

### Isolation of peripheral blood mononuclear cells (PBMCs)

Blood samples (20mL) were collected from the study population in EDTA K3 tubes and peripheral blood mononuclear cells (PBMCs) were isolated using the Ficoll density gradient centrifugation method as previously described by Katawa *et* al. 2015 [4]. In brief, whole blood was diluted in PBS and carefully added to 15mL of Pancoll (PAN Biotech, Aidenbach, Germany). Following 20 minutes centrifugation at 1300 rpm, the white layer of PBMCs was collected and washed 3 times in RPMI 1640 supplemented with 10% FBS (PAN Biotech). Cells were counted, and their viability was assessed using the trypan blue exclusion method.

### Preparation of hydro-ethanolic extracts

Fresh trunk barks of *Khaya senegalensis* were harvested in the forest of Tchavadè (Central region of Togo), grains of *Aframomum melegueta*, and fresh fruits of *Xylopia aethiopica* were bought on the market. These materials were rinsed and air-dried at laboratory conditions (18–25°C). Plant materials were powdered in the mill. Thereafter, 100 g of powdered plant materials were added to 500 mL of the ethanol-water mixture (70: 30) for 48 hours. The mixture was then filtered with Wathman filtrer paper and the filtrate was subsequently evaporated using a Rotary evaporator at 50°C. The extraction yield for each plant ranged from 19–22%.

To perform anti-inflammatory assay, 100mg/mL of extracts were prepared by adding 1g of evaporated plant materials to 10mL of distilled water. From this, a concentration of 200 μg/mL for each plant extract was prepared and filtered using 0.45μm millipore membrane.

The total flavonoid and phenolic from the same batch of hydro-ethanolic extracts compounds were characterized by our group [27] (S1 Table).

### Deglycosylation of hydro-ethanolic extracts

The plant extracts were deglycosylized using Glycoprotein Deglycosylation Kit (Merck KGaA, Darmstadt, Germany) according to manufacturer instructions. Briefly, to 30μL of hydro-ethanolic extracts at a concentration of 100 mg/mL, were added respectively 10 μl of 5X reaction buffer (250 mM sodium phosphate buffer, pH 7.0), 2.5 μL of denaturing solution (2% SDS, 1 M β-mercaptoethanol), 2.5 μL of the detergent solution (TRITON X-100), 1 μL of N-glycosidase F, 1 μL of α2–3,6,8,9-neuraminidase, 1 μL of endo-α-N-acetylgalactosaminidase, 1 μL of β1,4-galactosidase and 1 μL of β-N-Acetylglucosaminidase. Thereafter, the mixture was incubated at 37°C in a water bath for 24 hours and filtred with 0.45 μm sterile syringe filter (VWR, Puerto Rico, USA) for assays.

### Delipidation hydro-ethanolic extracts

Hydro-ethanolic extracts were delipidated with Lipid Removal Agent LRA (Sigma-Aldrich Chemie GmbH, Taufkirchen, Germany) according to the manufacturer instructions. In brief, 1 mL of hydro-ethanolic extracts at a concentration of 2 mg/mL was incubated with 1 mL of LRA successives contrations of 100 mg/mL for 3 hours at room temperature. After what, centrifugation was performed at 1500 rpm for 5 minutes, supernatants were recovered and filtred with 0.45 μm sterile syringe filter (VWR, Puerto Rico, USA) for assays.

### Deproteinization hydro-ethanolic extracts

An aliquot of 2 ml of each plant extract at 100 mg/mL was heated at 95°C in a water bath for 5 minutes, cooled and filtred with 0.45 μm sterile syringe filter (VWR, Puerto Rico, USA). There after 200 μg/mL was prepared for cell stimulation.

### *In vitro* cell culture

Isolated PBMCs from GEO, EN and HO individuals were cultured in RPMI 1640 (Life Technologies Corporation, USA) supplemented with 10% FBS (PAN Biotech, Aidenbach, Germany), gentamycin (50 μg/mL) (PAN Biotech, Aidenbach, Germany), penicillin/streptomycin (50 μg/mL) (PAN Biotech, Aidenbach, Germany), and L-glutamine (292.3 μg/ml) (GIBCO Thermo Fisher Scientific, New York, USA). Approximately, 2×10^5^ PBMCs/well were activated with αCD3/CD28 microbeads (Invitrogen, Carlsbad, California, USA) in the presence or absence of 200μg/mL of the hydro-ethanolic extracts. This concentration was chosen after an optimization step from 400 to 0.195ug/mL. The 200ug/mL was defined as concentration that do not induce IL-17A production (S1 Fig) with significant inhibitory activity upon TCR activation (S2 Fig) with less cell cytotoxicity (S3 Fig). In our previous study, we showed that 200 μg/mL of hydro-ethanolic was neither toxic nor cytotoxic [22,27]

Deglycosylated, delipidated and deproteinized fractions from hydro-ethanolic extracts were also used at 200 μg/mL after αCD3/CD28 stimulation. Plates were then incubated at 37°C under CO_2_ and humid atmosphere for 24 hours and 72 hours.

### Cytokine assessment

Levels of Th1 (IFN-γ), Th17 (IL-17A), Th2 (IL-5) and Treg (IL-10) cytokines were measured from cell culture supernatants by sandwich ELISA technique using the ready to use Invitrogen kit (Thermo Fisher Scientific, San Diego, USA) according to the manufacturer’s instructions. Cytokines’ concentrations were assessed using a HumaReader HS (Human, Wiesbaden, Germany).

### Immune cells profiling using flow cytometry

To characterize T helper cells after stimulation, cell culture pellet was harvested after 72 hours culture and stained as described by Katawa et al., 2015 [4]. Briefly, cells were activated with a Stimulation Cocktail containing Phorbol 12-myristate 13-acetate (PMA), Ionomycin and a protein transport inhibitor (Brefeldin A and Monensin) for 6 hours. Then, surface staining was performed using anti-human CD4-APC (clone: A161A1) for 30 minutes. After the fixation and permeabilization with 1/4x Fix-Perm reagent, the Fc block was added for 15 minutes to block the Fc receptor and unspecific antibody binding. Cells were then incubated at 4°C for 30 minutes with either 1) anti-human T-bet-PE (clone: 4B10) and IFN-γ-FITC (clone: 4S. B3) for Th1 cells, 2) GATA3-PE (clone: 16E10A3) and IL-4-FITC (clone: MP4-25D2) for Th2 cells, 3) RORC2-PE (clone: IC6006P) and IL-17A-FITC (clone: BL168) for Th17 cells or 4) Foxp3-FITC (clone: 206D) or IL-10-PE (clone: JES3-9D7) for regulatory T cells. Cells were finally washed twice and resuspended in Fix-Perm buffer.

To investigate the effects of hydro-ethanolic extracts on helper T cells activation and proliferation, cell pellets were harvested upon 24 hours of cell culture and stained using anti-human CD4-APC (clone: A161A1) in combination with either 1) CTLA4-FITC (clone: 14D3), CD69-PE (clone: FN50), 2) CD80-FITC (clone: 2D10), CD86-PE (clone: IT2.2), 3) PD1-PE (clone: 14D3) or 4) PCNA-PE (clone: PC10). All antibodies and reagents were obtained from Thermo Fischer (New York, USA)/eBioscience (San Diego, California, USA).

**Fig 2:**
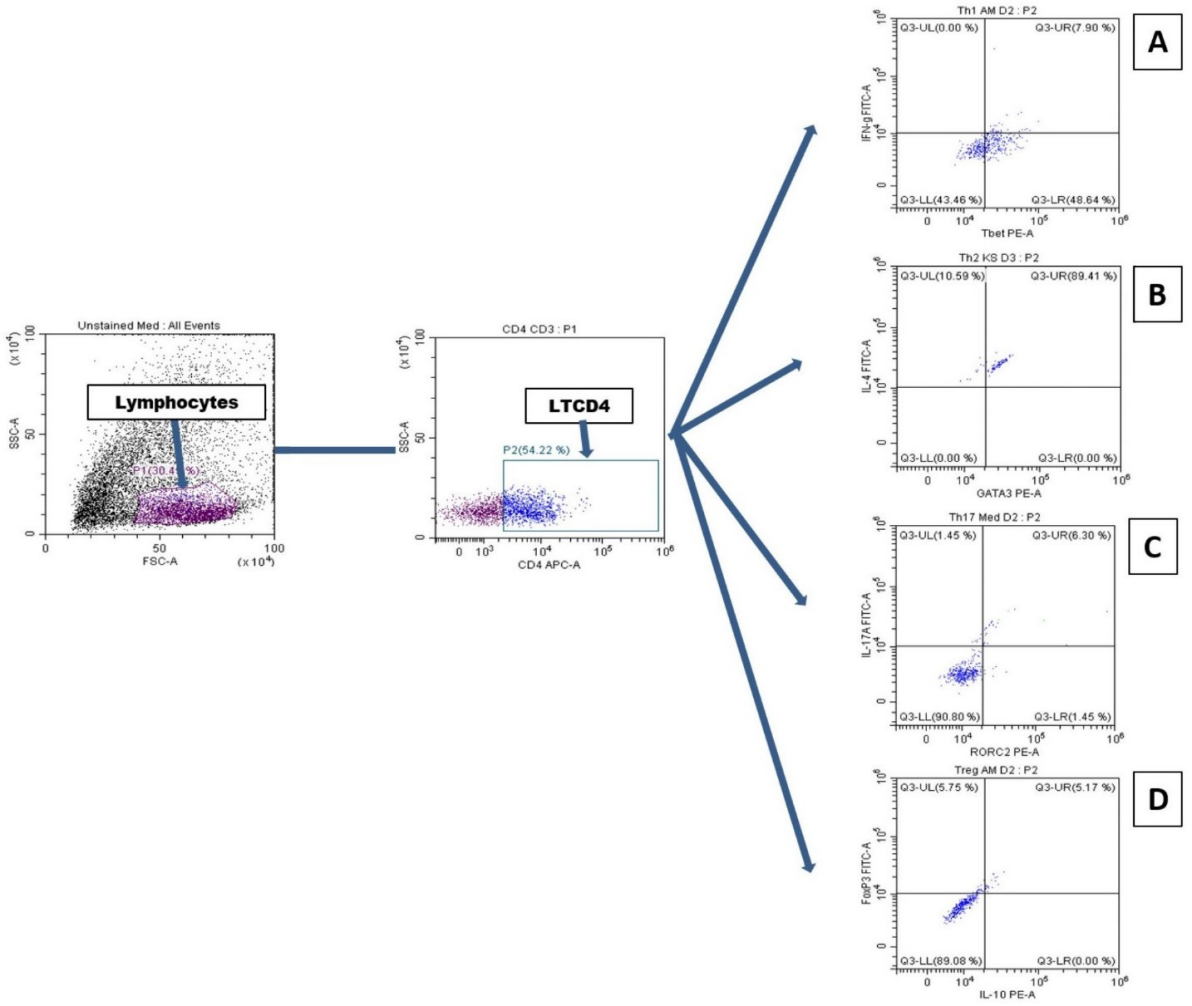
Gating strategy. Tbet^+^IFN-γ^+^ (A), GATA3^+^IL-4^+^ (B), RORC2^+^IL-17A^+^ (C) and Foxp3^+^IL-10^+^ (D) cells of CD4 cells were analyzed from lymphocytes gate. Positives and negatives controls are presented in S2 Table.

Cells were acquired on a Cytoflex Flow Cytometer (Beckman Coulter, Brea, Califinia, USA) and data were analyzed with CytExpert 2.1. (Beckman Coulter, Brea, Califinia, USA). Spectral overlap was corrected by performing fluorescence compensation using VersaComp Antibody Capture Bead Kit (Beckman Coulter, Brea, Califinia, USA) and fluorescence minus one (FMO) control were acquired to discriminate populations. The gating strategy is shown in Fig 2. Positive and negative are presented in S2 Table and representative plots for the main conditions are in S3 Table.

### Statistical analysis

Statistical analyses were performed using the software GraphPad PRISM 5.02 for Windows (GraphPad Software, Inc., La Jolla, USA). Depending on the distribution parameter of the data sets, statistical differences were observed using Kruskal-Wallis-test and, if significant, followed by Mann-Whitney-U test for a further comparison of two groups. P-values of 0.05 or less were considered significant * p<0.05, ** p<0.01 and *** p<0.01.

## Results

### Sociodemographic characteristics and epidemiology of onchocerciasis

In total, 237 individuals were enrolled in this study comprising generalized onchocerciasis (GEO, n = 23), hyperreactive onchocerciasis (HO, n = 6) and infection-free volunteers, residing in the same area (EN, n = 208). However, whereas the mean age was similar in the study groups, males were significantly more represented and individuals in the EN group lived longer in the study site compared to the GEO and HO group. Table 1 summarizes the characteristics of the study population.

**Table 1:** Characteristics of the study population.

Characteristics	EN	GEO	HO
Age (Years)	35.94±11.67	43.47±11.57	52.60±4.278
Male *n* (%)	111 (53.37)	19 (82.61)	3 (50)
Female *n* (%)	97 (46.63)	4 (17.39)	3 (50)
Life in locality ˃ 10 years *n* (%)	207 (88.84)	21 (9.01)	5 (2.15)
Last intake of ivermectin ˃ 1 year *n* (%)	3 (1.27)	0 (0)	0 (0)
Palpable onchocercal nodules *n* (%)	0 (0)	17 (94.44)	1 (5.56)
MF *n* (%)	0 (0)	5 (71.43)	2 (28.57)
DPM *n* (%)	0 (0)	0 (0)	2 (100)
Total *n* (%)	208 (87.76)	23(9.70)	6(2.53)

DPM: depigmented leopard skin; EN: endemic normal; GEO: generalized onchocerciasis; HO: hyperreactive onchocerciasis.

### Hydro-ethanolic extracts of *Aframomum melegueta*, *Khaya senegalensis* and *Xylopia aethiopica* downregulated IL-5 and IL-17A cytokines induction in HO individuals

**Fig 3:**
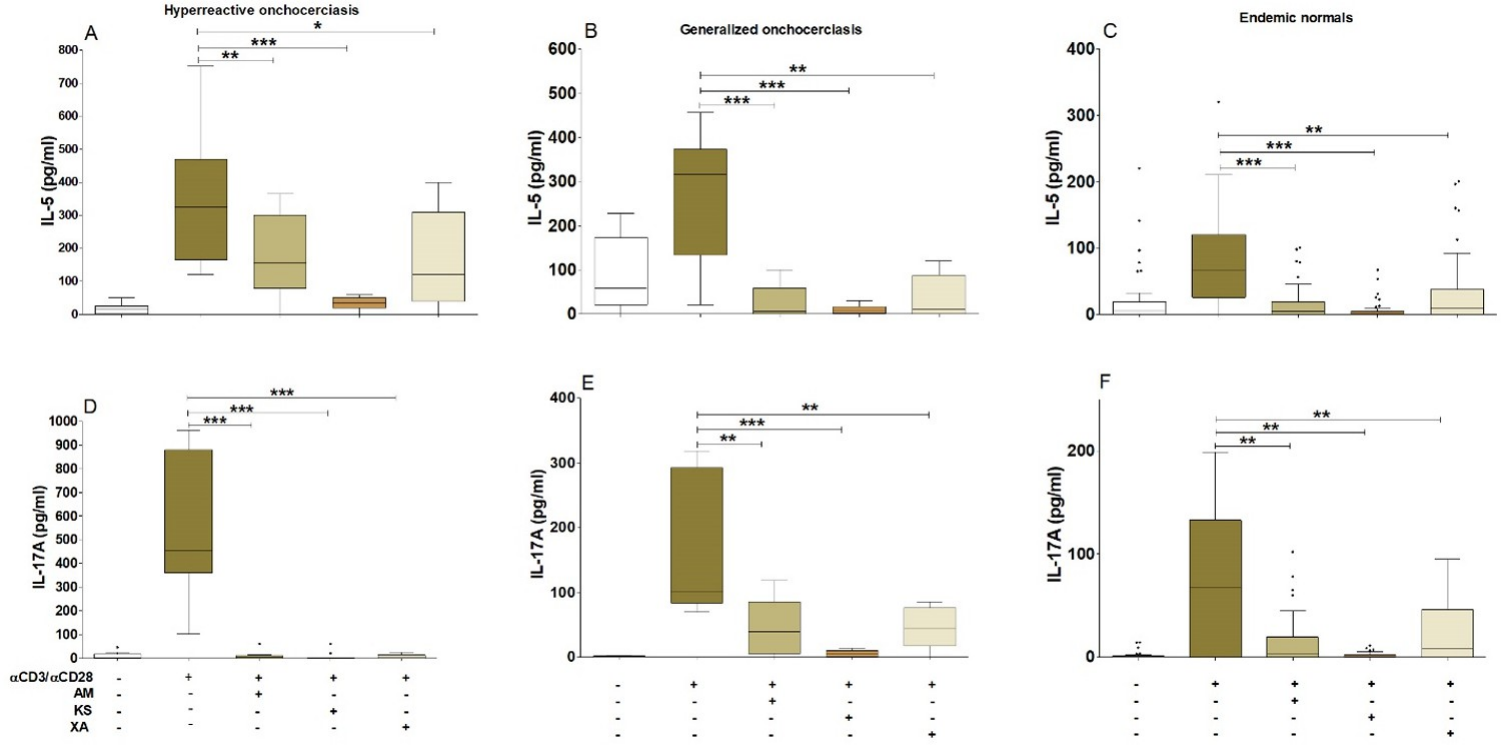
Inhibition of IL-17A and IL-5 induction in HO, GEO and EN individuals by the hydro-ethanolic extracts of *Aframomum melegueta*, *Khaya senegalensis* or *Xylopia aethiopica*. PBMCs (2x105 cells) isolated from EN (n = 13), GEO (n = 15) and HO (n = 6) individuals were activated with αCD3 (12.5 μg /well) / αCD28 (2.5 μg / well) in the presence or absence of *Aframomum melegueta* (AM), *Khaya senegalensis* (KS) or *Xylopia aethiopica* (XA) extracts (200 μg / mL). Box whiskers (tukey) with outliers show levels of IL-5 (A, B and C) and IL-17A (D, E and F) after co-culture with αCD3 / αCD28 in presence or absence of AM, KS or XA. Asterisks show statistical differences (Mann-Whitney-U test) between the groups indicated by the brackets (*p<0.05, **p<0.01, ***p<0.001).

**Fig 4:**
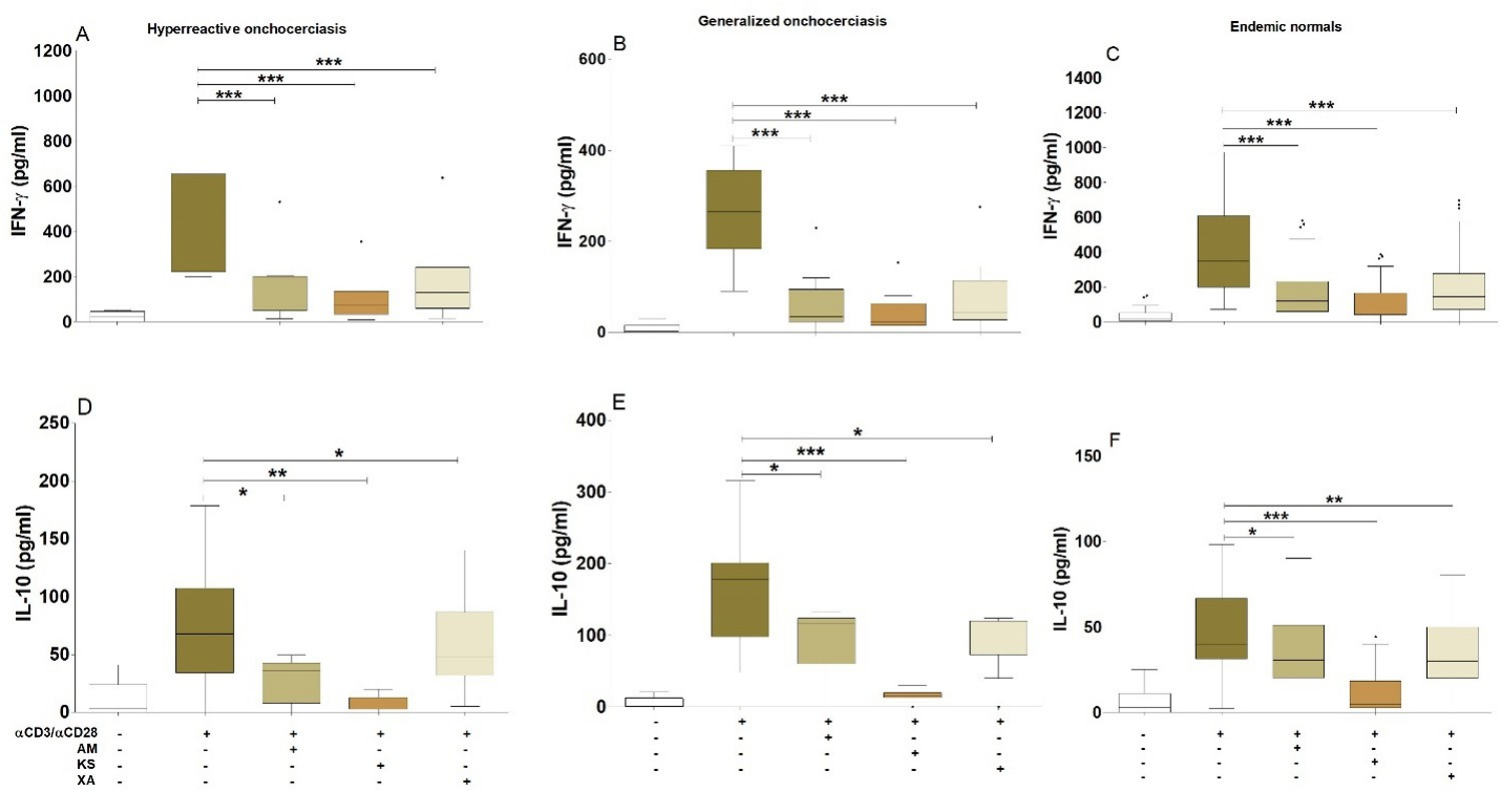
Inhibition of IFN-γ and IL-10 induction by the hydro-ethanolic extracts of *Aframomum melegueta*, *Khaya senegalensis* or *Xylopia aethiopica*. PBMCs (2x105 cells) isolated from EN (n = 13), GEO (n = 15) and HO (n = 6) individuals were activated with αCD3 (12.5 μg /well) / αCD28 (2.5 μg / well) in the presence or absence of *Aframomum melegueta* (AM), *Khaya senegalensis* (KS) or *Xylopia aethiopica* (XA) extracts (200 μg / mL). Box whiskers (tukey) with outliers show levels of IFN-γ (A, B and C) and IL-10 (D, E and F) after co-culture with αCD3 / αCD28 in presence or absence of AM, KS or XA. Asterisks show statistical differences (Mann-Whitney-U test) between the groups indicated by the brackets (*p<0.05, **p<0.01, ***p<0.001).

HO individuals produced elevated concentrations of IL**-**5 (Fig 3A–3C) and IL-17A (Fig 3D–3F) upon TCR activation compared to unstimulated controls. This production of IL-17A in HO individuals was high compared to EN and GEO individuals (Fig 3). The activation of TCR in the presence of *Aframomum melegueta*, *Khaya senegalensis* or *Xylopia aethiopica* significantly abrogated TCR-induced IL-17A and IL**-**5 production. The inhibitory activity of plants’ extracts on IL-17A induction was more augmented in HO individuals (Fig 3D) compared to GEO (Fig 3E) and EN (Fig 3F). This could indicate an anti-inflammatory (Th17) and an anti-Th2 properties of the hydro-ethanolic fraction of these plants. We then aimed to investigate whether these extracts use Th1 or Treg pathways. Therefore, INF-γ and IL-10 were measured in the same cell culture supernatants. Surprisingly, we observed that INF-γ (Fig 4A–4C) and IL-10 (Fig 4D–4F) were also inhibited by the hydro-ethanolic extracts of *Aframomum melegueta*, *Khaya senegalensis* or *Xylopia aethiopica*, showing that the inhibitory pathway of IL-17A and IL**-**5 by these extracts is not INF-γ and IL-10-dependent.

### *Aframomum melegueta* and *Xylopia aethiopica* inhibited CD4^+^RORC2^+^IL-17A^+^ and CD4^+^GATA3^+^IL4^+^ cell populations induction

**Fig 5:**
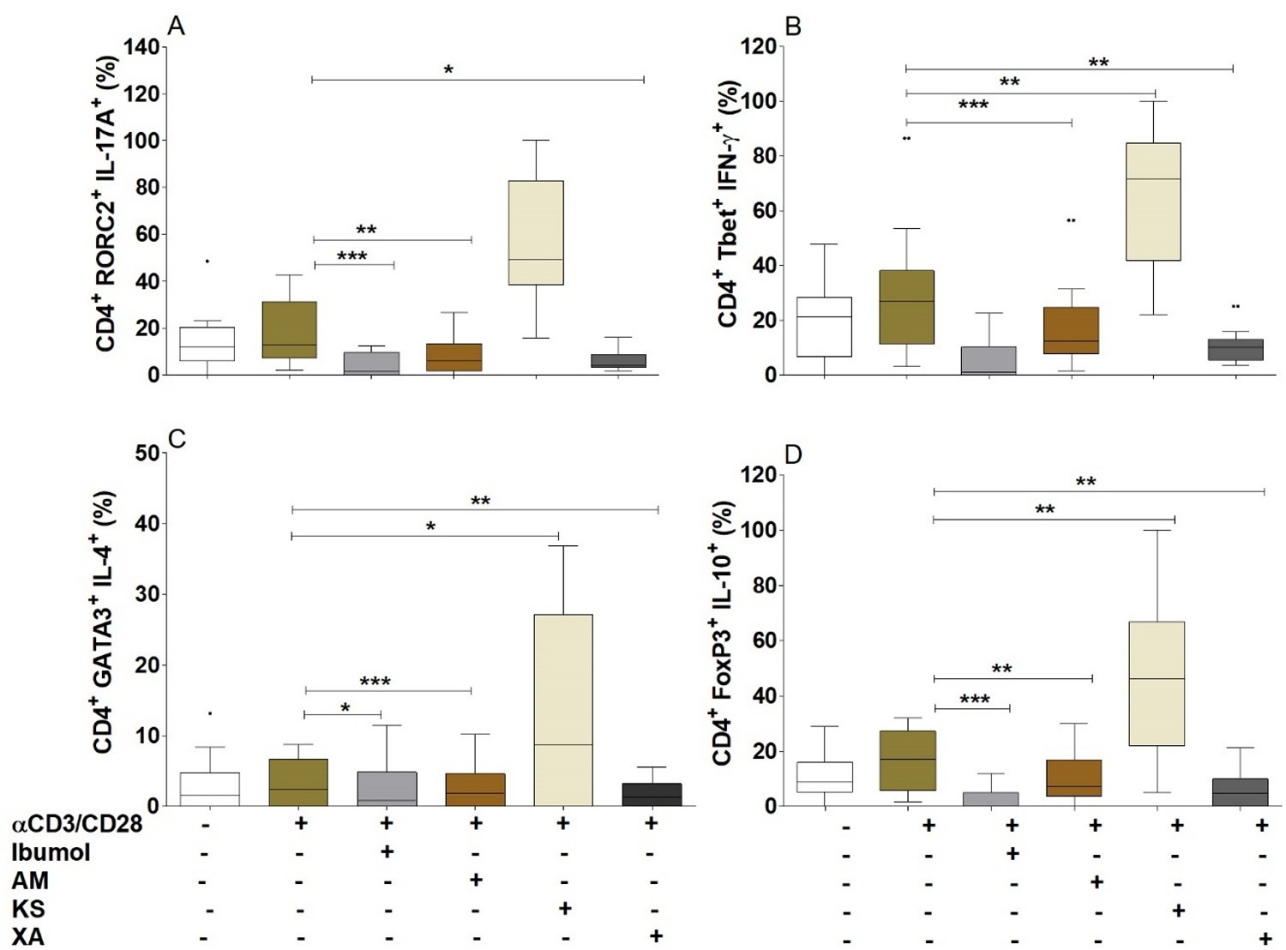
Effect of the plants’ hydro-ethanolic extracts on T helper cells populations. PBMCs (2x10^5^ cells) from endemic normal individuals (n = 9) were activated with αCD3/αCD28 (0.5 beads/μl) in the presence or absence of Ibumol, *Aframomum melegueta* (AM), *Khaya senegalensis* (KS) or *Xylopia aethiopica* (XA) (200 μg / ml) for 3 days. Thereafter, cells were activated with Phorbol Myristate Acetate (PMA) for 6 hours and stained for CD4, Th specific cytokines and transcription factors: Th17 (A), Th1 (B), Th2 (C) and Tregs (D). Graphs show percentages as box whiskers (tukey) with outliers. Asterisks show statistical differences (Mann-Whitney-U test) between the groups indicated by the brackets (*p<0.05, **p<0.01, ***p<0.001).

From cell culture supernatants, we observed that the hydro-ethanolic extracts of *Aframomum melegueta* and *Xylopia aethiopica* inhibited IFN-γ, IL-17A, IL-5 and IL-10 production. Here, we aimed to investigate the effect of these extracts on T cell phenotype by flow cytometry. Therefore, T cells’ signature cytokine and their respective transcription factors were analyzed from cell pellets upon TCR activation in the presence or absence of plants hydro-ethanolic extracts. Fig 5 shows that the hydro-ethanolic extracts of *Aframomum melegueta* and *Xylopia aethiopica* inhibited the development of CD4^+^RORC2^+^IL-17A^+^ (Fig 5A), CD4^+^Tbet^+^IFN-γ^+^ (Fig 5B), CD4^+^ GATA3^+^ IL4^+^ (Fig 5C) and CD4^+^FoxP3^+^IL-10^+^ (Fig 5D) cell populations while *Khaya senegalensis* supported their induction.

### *Khaya senegalensis* but not *Aframomum melegueta* nor *Xylopia aethiopica* inhibited T Cells activation and proliferation

**Fig 6:**
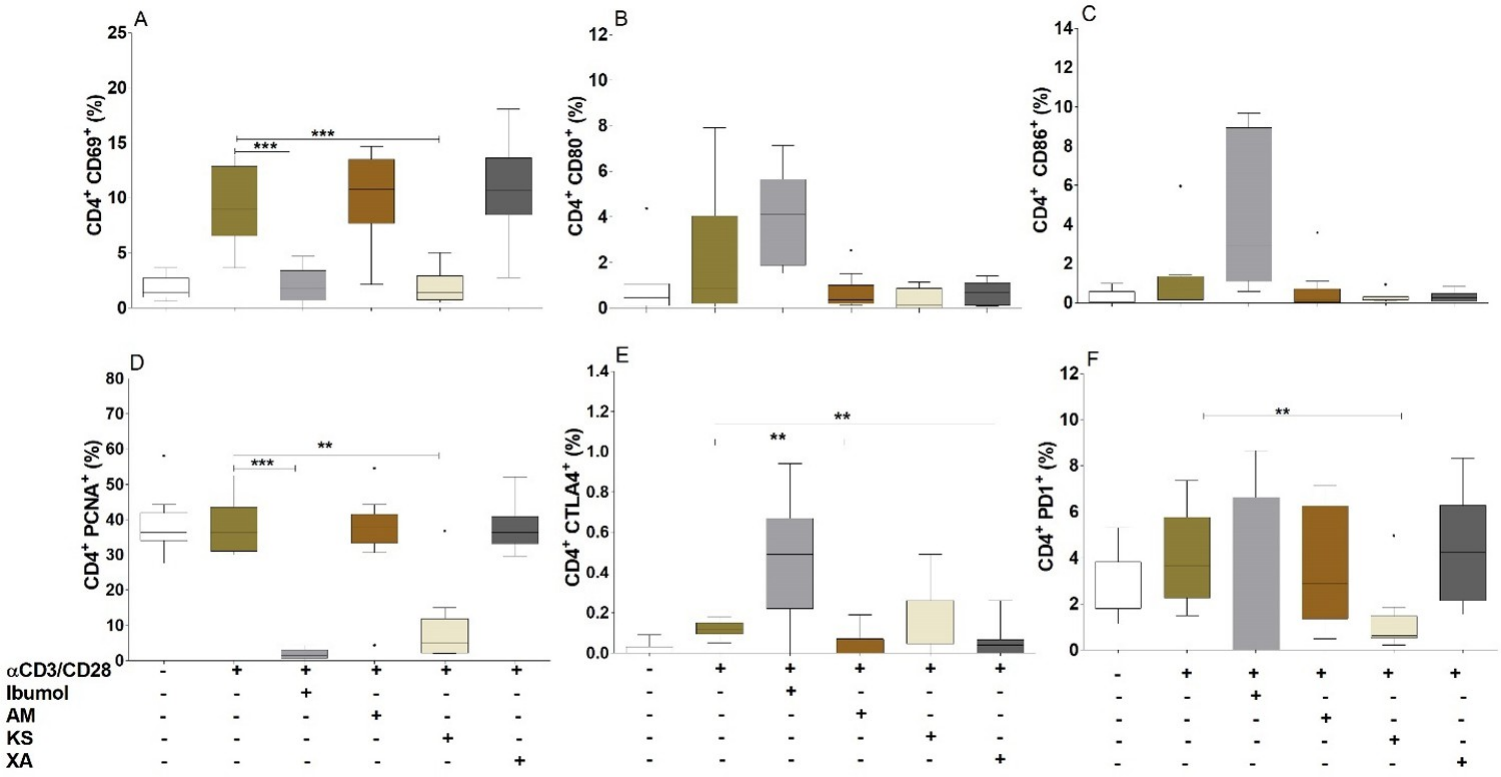
T cells activation and proliferation by *Aframomum melegueta*, *Khaya senegalensis* and *Xylopia aethiopica* hydro-ethanolic extracts. PBMCs (2x10^5^ cells) from endemic normal individuals (n = 9) were activated with αCD3/αCD28 (0.5 beads/μl) in the presence or absence of Ibumol, *Aframomum melegueta* (AM), *Khaya senegalensis* (KS) or *Xylopia aethiopica* (XA) (200 μg / ml) for 24 hours. Thereafter, cells were stained for CD4 in combination with either CD69 (A), CD80 (B), CD86 (C), PCNA (D), CTLA4 (E) or PD1 (F). Graphs show percentages as box whiskers (tukey) with outliers. Asterisks show statistical differences (Mann-Whitney-U test) between the groups indicated by the brackets (*p<0.05, **p<0.01, ***p<0.001); *ns* (not significant).

We got to know if plants’ hydro-ethanolic extracts interfere with T cells activation and proliferation. Briefly, the expression of CD4^+^CD69^+^ (Fig 6A) and CD4^+^PCNA^+^ (Fig 6D) were significantly inhibited by *Khaya senegalensis*. In contrast, CD80 (Fig 6B) and CD86 (Fig 6C) were not inhibited by these three plant extracts. In addition, *Aframomum melegueta* and *Xylopia aethiopica* significantly inhibited the expression of CTLA-4 (Fig 6E), while PD-1 (Fig 6F) was inhibited by *Khaya senegalensis*.

### Deproteinization and delipidation of *Aframomum melegueta* or *Xylopia aethiopica* hydro-ethanolic extracts abrogated their IL-17A inhibition properties

**Fig 7:**
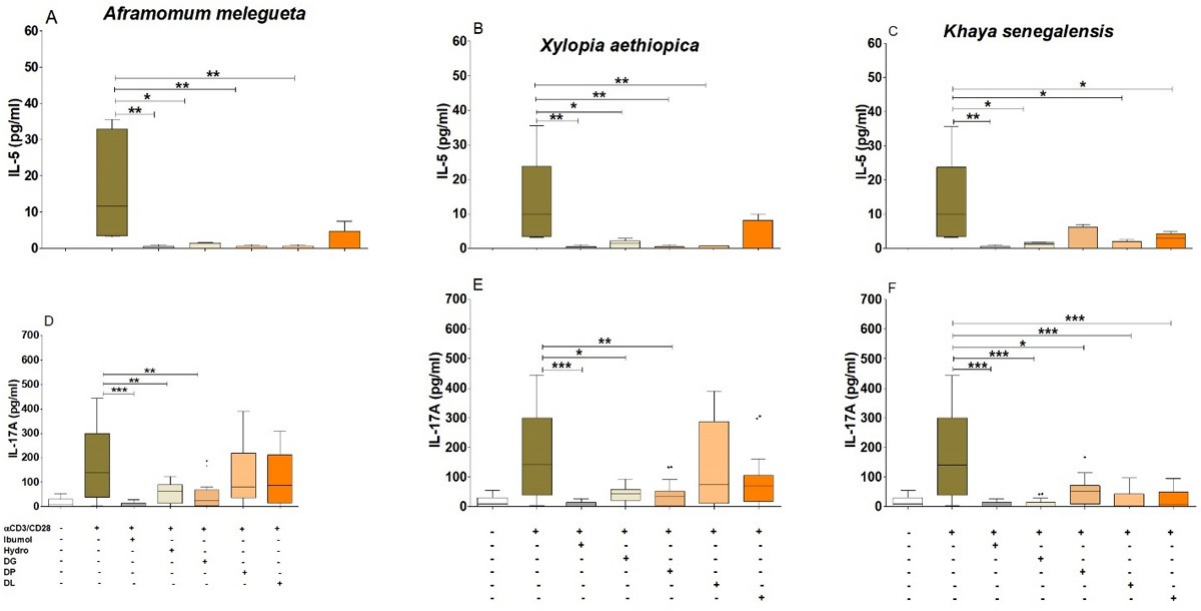
Effect of deglycosylation, deproteinization or delipidation of the hydro-ethanolic extracts of *Aframomum melegueta*, *Khaya senegalensis* and *Xylopia aethiopica*. PBMCs (2x105 cells) from endemic normal individuals (n = 16) were activated with αCD3/αCD28 (0.5 beads/μl) in the presence or absence of Ibumol, hydro-ethanolic extracts (Hydro), deglycosylated (DG), deproteinized (DP) or delipidated (DL) fractions of *Aframomum melegueta* (AM), *Khaya senegalensis* (KS) and *Xylopia aethiopica* (XA) (200 μg / ml) for 3 days. Box whiskers (tukey) with outliers show levels of IL-5 (A, B and C) and IL-17A (D, E and F). Asterisks show statistical differences (Mann-Whitney-U test) between the groups indicated by the brackets (*p<0.05, **p<0.01, ***p<0.001).

Since the inhibition of the cytokines IL-17A and IL-5 by the hydro-ethanolic extracts of *Aframomum melegueta*, *Khaya senegalensis* and *Xylopia aethiopica* do not use Th1 nor Treg pathways, we lastly wanted to know if the anti-inflammatory (Th17) and anti-Th2 responses observed could depend on the activity of the major chemical components present in these extracts. To this end, hydro-ethanolic extracts of these plants were deproteinized, delipidated or deglycosylated for *in vitro* stimulation of PBMCs from EN. The results showed a differential effect of deglycosylation, deproteinization and delipidation on the inhibition properties of the hydro-ethanolic extracts of *Aframomum melegueta*, *Khaya senegalensis* or *Xylopia aethiopica*. In brief, the deglycosylated fraction of *Aframomum melegueta* had no effect on the inhibition of the induction of IL-5 (Fig 7A) and IL-17A (Fig 7D) by the hydro-ethanolic extracts. Deproteinized and delipidated fractions of *Aframomum melegueta* and *Xylopia aethiopica* abrogated the inhibition of IL-17A production (Fig 7D and 7E). When delipidated, hydro-ethanolic extracts of the three plants failed to abrogate the inhibition of IL-5 (Fig 7A–7C). Deproteinization of hydroethanolic extracts of *Khaya senegalensis* did not abrogate their inhibition properties of IL-5 (Fig 7C) and IL-17A (Fig 7F).

## Discussion

Unprecedented progress has been made in reducing NTDs over the past decade, successes which WHO looks forward to continuing in the years to come [28]. The fight against these NTDs including onchocerciasis in Africa since 1974 has been one of the most successful activities in health and development in terms of achievements in public health, in partnership with social and economic development [8]. Despite this success of the WHO, Africa and specifically Togo is not yet spared from onchocerciasis. Onchocerciasis is still endemic in few villages of Togo, mainly, in the Central and Kara regions [22]. This study was undertaken in 6 villages (Bato, Takadè, Kouida, Tchetchekou, Tchatoukoura and Banda) of the “Préfecture de Mô” in the Central region of Togo. The prevalence of onchocerciasis was about 12.24% (29/237 subjects). Males were more infected than female individuals and the median age of subjects having a form of onchocerciasis was over 30 years (43.47 years for women and 52.60 for men). Disease profiles vary by geographic region and sex with higher rates of microfilariderma and morbidity in men than in women [7,29]. Men are more represented because of the rural activities in these fertile basins where they are constantly exposed compared to women. They go to the field early and come back home late and that may explain this male prevalence. To this male prevalence, we can also glue the capacity of the weak immune response to this chronic parasitic infection because, women with chronic inflammatory pathologies have a much stronger immune response compared to men by the advantage of one more X chromosome than men [30]. On the other hand, other authors have associated these variations with the heterogeneity of age and sex on exposure to vectors [31]. This result confirms the data provided in the report on the establishment of the specific committee of experts on onchocerciasis in 2016 of the National Onchocerciasis Control Program (PNLO) in Togo by the Ministry in charge of health, according to which there are still areas of Togo where the prevalence of the disease is still above 5%, especially in the basins of Kéran (14.8% in 2014), Amou (10% in 2013) and Mô (26.5% in 2014) [32]. The median prevalence of *O*. *volvulus* infections dropped below 5% in 2014, but in several locations the MF-positivity exceeded this level in the river basins of Ôti, Kéran and Mô [29]. This may be linked to a low coverage rate for mass drug administration with ivermectin since in 2016, this rate was 54%, i.e. 3.8 million of Togolese benefited from the treatment while those not having benefited were estimated at 2.5 million [33]. Ivermectin has been used for a decade for the treatment of severe form of onchocerciasis by the African Programme for Onchocerciasis Control (APOC) [29]. Severe form onchocerciasis is characterized by skin inflammation and dermatitis [3]. Ivermectin is a microfilaricide drug and has no anti-inflammatory properties. This study was undertaken, to investigate new drug approach with both microfilaricide and anti-inflammatory properties. It is known that endemic communities used traditional medicine approach to treat disease [34]. To this end, an ethnobotanical survey was carried out in the endemic area of ​​the Kara and Central regions of Togo. The data revealed the use of medicinal plants to treat helminthiasis [22]. The most used plants were *Aframomum melegueta*, *Xylopia aethiopica* and *Khaya senegalensis* [22]. The formulation of the recipes was mostly decoction and the oral administration route was more used [22]. Our group demonstrated *in vitro* the microfilaricide effects of the hydro-ethanolic extracts of these most cited plants by the traditional healers to treat helminth infections [22]. In this second part of the project, we aimed to investigate the anti-inflammatory properties of these plants. The limit of this study is that GEO and HO individuals became rare in the communities, so the sample size of this cohort was small.

Hyperreactive onchocerciasis is characterized by skin damage and dermatitis. The disease severity was shown to be Th17 and Th2 dominant [4]. Our data showed a significant inhibition of the production of the inflammatory cytokine (IL-17A) and anti-Th2 cytokine (IL-5) in PBMCs from EN, GEO and HO subjects co-cultured with hydro-ethanolic extracts of *Aframomum melegueta*, *Khaya senegalensis* or *Xylopia aethiopica*.

Many studies focused on the anti-inflammatory effects of plants extracts but few used our *in vitro* setup approach [35,36]. The anti-inflammatory activity of the ethanolic extract of the seeds of *Aframomum melegueta* was studied by Ilic et al. in 2014 in a murine model and in pro-inflammatory monocyte-macrophage gene expression assays. To this end, they obtained an inhibition rate of 76% of cyclo-oxygenase 2 (COX-2) with a concentration of 1 mL/kg of *Aframomum melegueta* extract against 78% inhibition rate for the positive control (Viox). The crude extract also reduced inflammation by 49% with 100 mL/kg of extract [37]. This property has been attributed to the inhibition of COX-2 and also to the inhibition of the IL-1-gene, a pro-inflammatory gene [37]. The hydro-ethanolic extracts of the fruits of *Xylopia aethiopica*, on the other hand, inhibit immediate allergic reactions dependent on mast cells and exhibit anti-inflammatory actions by inhibiting the release of histamine by mast cells by stabilizing the cell membrane [38]. Evaluation of TNF-α levels in THP-1 macrophages caused by LPS showed a significant inhibition (> 90%) during treatment with the hydro-ethanolic extract of the leaves of *Xylopia aethiopica* at a concentration of 500 μg / mL. Additional anti-inflammatory effects were recorded, including a significant decrease in IL-6 levels at 250 μg / mL and 500 μg / mL and a significant inhibition of lipoxygenase at concentrations ranging from 16 μg / mL to 250 μg / mL [39]. Also, pro-inflammatory mediators such as IL-8, IL-6 and the enzyme cyclo-oxygenase 2 (PTGS2) (COX-2) were significantly inhibited [40]. It is demonstrated that the anti-inflammatory activity of *A*. *melegueta* seeds was due to the major bioactive molecules of these grains such as gingerol, shogaol and -paradol [6]. These molecules are among the compounds of the gingerol family that are the inhibitors of the arachidonic acid synthesis pathway [37]. Also, the oral administration of 1000 mg/kg and 1500 mg/kg of aqueous extract of the mixture *Afromomum melegueta-Citrus aurantifolia* to guinea pigs has shown not only anti-inflammatory effects but also, anti-edematous, analgesic and antipyretics effects. In our study IL-10 and INF-γ were also inhibited by the plants extracts indicating that the inhibition pathway of IL-17A and IL-5 is not Th1 and/or IL-10 dependent.

After the optimization of the plant extracts concentration to be used, we previously showed that 200 ug/mL had no cytotoxicity, no acute and no sub-chronic toxicity in Wistar rats [22] and our previous study demonstrated that *Aframomum melegueta*, *Khaya senegalensis and Xylopia aethiopica* are three major plants, with microfilaricide effects, used in the Central and Kara regions of Togo by the traditional healers to treat onchocerciasis [22].

These biological properties observed depends on the activity of the major chemical components present in *Aframomum melegueta* and *Xylopia aethiopica*, including alkaloids, gall tannins, flavonoids, triterpenoids, anthocyanosides, leuco-anthocyanins, mucilage and other compounds. Since the inhibitory effects observed could rise from lipid, carbohydrates, or proteins fractions, we proceeded to the delipidation, deglycolization and deproteinization of our hydro-ethanolic extracts. Deproteinizated and delipidated fractions of *Aframomum melegueta* and *Xylopia aethiopica* hydro-ethanolic extracts abrogated their IL-17A inhibition. This means that the major components in these two plants that inhibit IL-17A production could be of protein or lipid nature. Likewise, a significant inhibition of IL-17A production was obtained in a study performed on a mouse model with lupus, a chronic inflammatory disease with high production of IL-17A and treated with aconitine (alkaloid) [41]. However, Yatoo et al. in 2007, found that the common compounds present in plants with anti-inflammatory properties are steroids, glycosides, phenolics, flavonoids, alkaloids, polysaccharides, terpenoids, cannabinoids, and fatty acids [42]. So, the phyto-compounds with anti-inflammatory properties can be of carbohydrate, protein, and lipid nature. Specifically, they may be studied in the suppression of IL-17A by the plant extracts. The main phenolic constituent of *Xylopia aethiopica*, kaempferol-3-O-rutinoside significantly contributed to the anti-inflammatory effects, notably through the inhibition of lipo-oxygenase. However, no effect on the decrease in TNF-α and IL-6 levels caused by this phenolic compound was found [43]. The phytochemistry of the bark of *Khaya senegalensis* reveals the presence of polyphenolic compounds, saponins, anthracene derivatives and steroids, fatty acids, carotenoids, coumarins, reducing compounds, flavonoids, carbohydrates, tannins, glycosides, sterols and triterpenes [44,45]. This may be the reason why the treatment of *Khaya senegalensis* extract did not have any effect on the inhibition of production of IL-17A. In general, secondary metabolites are recognized by their biological activities [46]. Thus, sterols and triterpenes are responsible for the anti-inflammatory activity of *Khaya senegalensis*. Traore, in 2006 had rather linked this property to triterpene derivatives and coumarins [47]. The inhibition of cytokine production has led to the characterization of PBMCs of normal endemic onchocerciasis after culturing these activated cells in the absence or presence of plant extracts for three days. It appears that these plants *Aframomum melegueta* and *Xylopia aethiopica* inhibit the immune responses of the Th17 and Th2 by a decrease in the expression of their transcription factors RORC2 and GATA3 respectively. A study carried out on gastric epithelial cells in Cameroon showed that *Aframomum melegueta* and *Xylopia aethiopica* both have anti-inflammatory properties following inhibition of the transcription factor NF-κB [40]. In general, we observed that *Khaya senegalensis* stimulatory conditions up-regulated T helper cells transcription factors. The cells were viable and this could be a biological activity that need to be explored. We aimed to undertake another study in order to study the impact of *Khaya senegalensis* extracts on Th cells transcription factors. Secondary metabolites not only act on soluble mediators of inflammation such as cytokines, chemokines, and the arachidonic acid pathway, but also on transcription factors, the recruitment of inflammatory cells. Specifically, flavonoids inhibit the cyclo-oxygenase pathway and the lipo-oxygenase pathway [48]. The mechanism of action of the suppression of the pro-inflammatory response by phyto-constituents is the inhibition of the PI3K / Akt, IKK / MAPK, mTORC1 pathways, the inhibition of NF-κΒ and JAK / STAT [49,50]. Different mechanisms could explain the inhibition properties of the plants. We therefore questioned whether these three plants regulated T cell function by inducing T cell suppressive markers such as CTLA-4 or PD-1 [51]. None of the plant extracts upregulated the expression of CTLA-4 and PD-1 molecules on T helper cells upon co-culture with PBMCs. Interestingly only *Khaya senegalensis* inhibits the activation and proliferation of cells. This indicated that, these plant extracts did not interfere with T cell function regulating markers. Antiproliferative activity of *Khaya senegalensis* bark extract HT-29, HCT-15 and HCA-7 cells was demonstrated [52].

## Conclusion

This study showed initial evidence that the hydro-ethanolic extracts from *Aframomum melegueta*, *Khaya senegalensis* and *Xylopia aethiopica* had anti-Th17 and ant-Th2 responses effects. Both AM and XA did not interfere with CD4^+^ cells activation and proliferation. These effects could be associated with protein or lipid fractions. Further fractionation and characterization of the hydro-ethanolic extracts will be performed to identify molecules carrying these effects.

## Supporting information

S1 Fig Optimization: Cytokines induction by different concentrations of hydro-ethanolic extracts. (TIF)

S2 Fig Optimization: IL-17A Inhibition of different concentration of hydro-ethanolic extracts of AM, KS and XA upon TCR activation. (TIF)

S3 Fig Optimization: Cytotoxicity of different concentration of hydro-ethanolic extracts of AM, KS and XA. (TIF)

S1 Table Characterization of flavonoid and phenolic compounds. (DOCX)

S2 Table Positives and negatives controls. (DOCX)

S3 Table Representative plots for the main conditions. (DOCX)
