# Supplementary figures and images for: Anti-Th17 and anti-Th2 responses effects of hydro-ethanolic extracts of Aframomum melegueta, Khaya senegalensis and Xylopia aethiopica in hyperreactive onchocerciasis individuals’ peripheral blood mononuclear cells
Source: PLoS Negl Trop Dis. 2022 Apr 25;16(4):e0010341. doi: 10.1371/journal.pntd.0010341 (PMC9071127; doi:10.1371/journal.pntd.0010341)

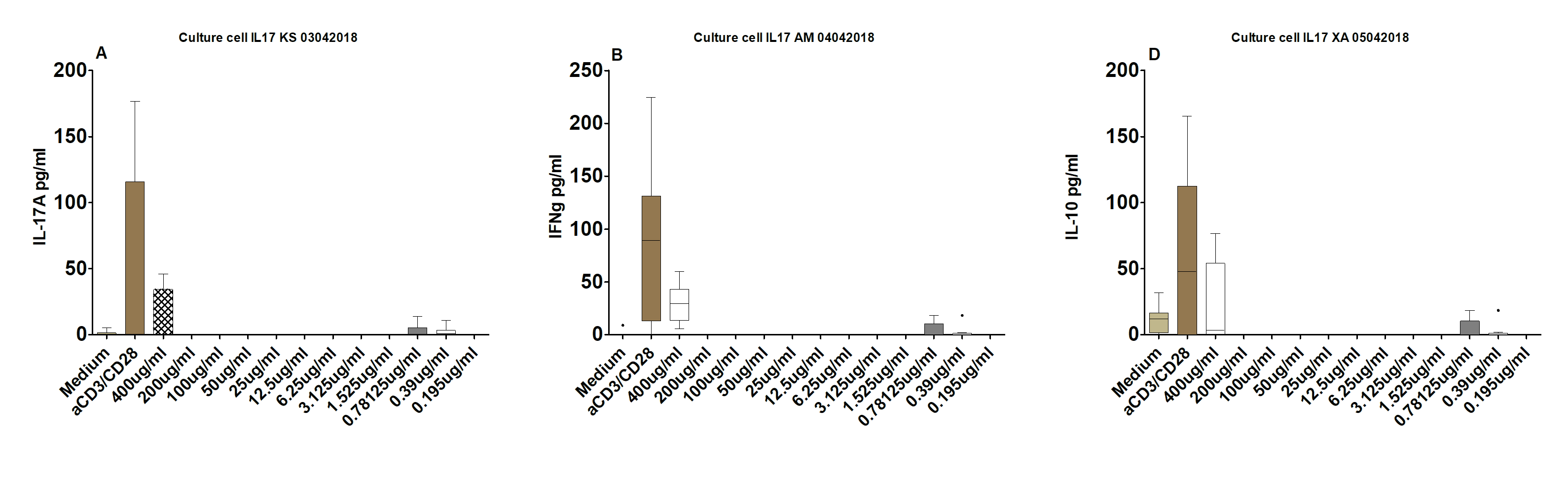

Supplement: S1 Fig — (TIF) [file pntd.0010341.s001.tif]

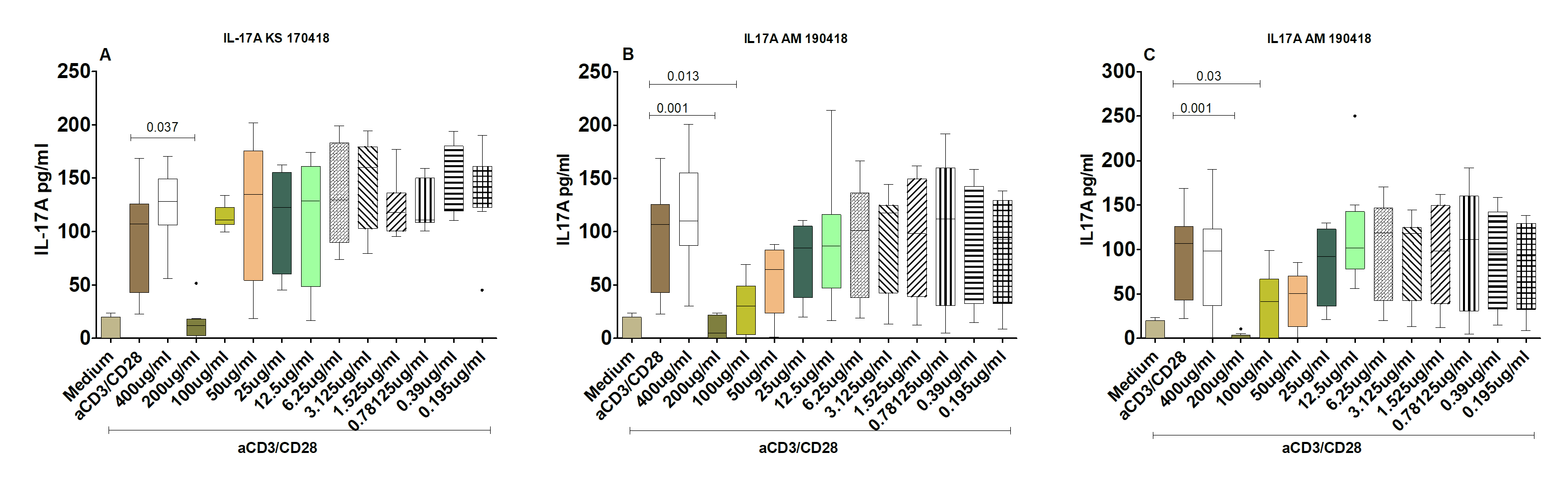

Supplement: S2 Fig — (TIF) [file pntd.0010341.s002.tif]

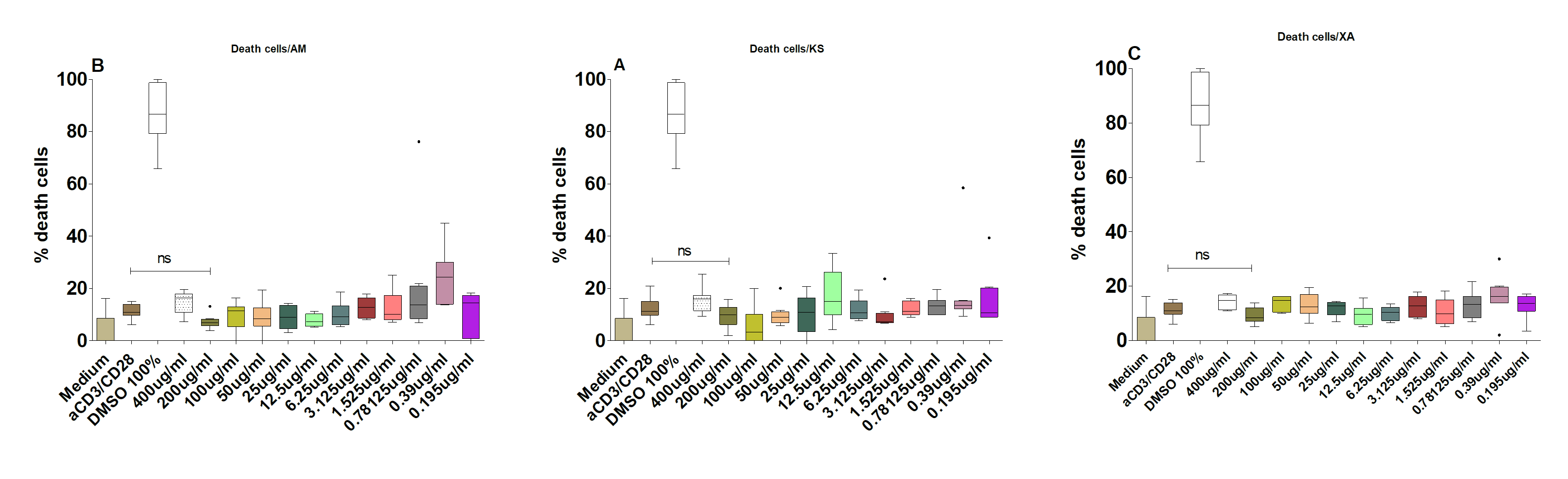

Supplement: S3 Fig — (TIF) [file pntd.0010341.s003.tif]
