# Supplementary material for: Anti-Th17 and anti-Th2 responses effects of hydro-ethanolic extracts of Aframomum melegueta, Khaya senegalensis and Xylopia aethiopica in hyperreactive onchocerciasis individuals’ peripheral blood mononuclear cells
Source: PLoS Negl Trop Dis. 2022 Apr 25;16(4):e0010341. doi: 10.1371/journal.pntd.0010341 (PMC9071127; doi:10.1371/journal.pntd.0010341)
Supplement: S1 Table — (DOCX) [file pntd.0010341.s004.docx]

**S1_Table** : Caracterization of Flavonoid and phenolic compounds

| **Extracts** | **Total phenols (mg EGA /100mg)** | **Total flavonoids (mg EQ/100mg)** |
| --- | --- | --- |
|  |  |  |
| ***K. senegalensis*** | 329.21±19.99 | 1.39±0.07 |
| ***A. melegueta*** | 174.45±14.62 | 1.02±0.05 |
| ***X. aethiopica*** | 15.27±1.09 | 0.52±0.04 |
