# Supplementary material for: Anti-Th17 and anti-Th2 responses effects of hydro-ethanolic extracts of Aframomum melegueta, Khaya senegalensis and Xylopia aethiopica in hyperreactive onchocerciasis individuals’ peripheral blood mononuclear cells
Source: PLoS Negl Trop Dis. 2022 Apr 25;16(4):e0010341. doi: 10.1371/journal.pntd.0010341 (PMC9071127; doi:10.1371/journal.pntd.0010341)
Supplement: S2 Table — (DOCX) [file pntd.0010341.s005.docx]

| **S2_Table** | Negative control/Unstained | Positive control/Single stained |
| --- | --- | --- |
| CD4-APC | 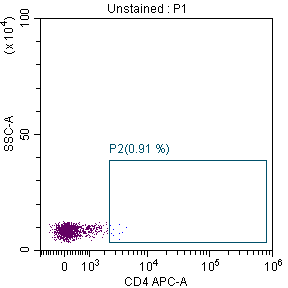 | 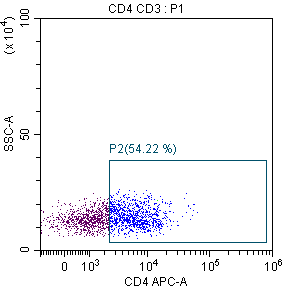 |
| IFNg-FITC | 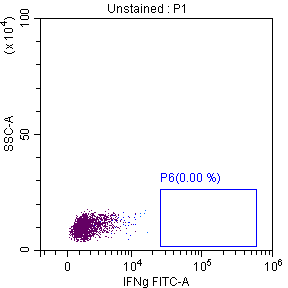 | 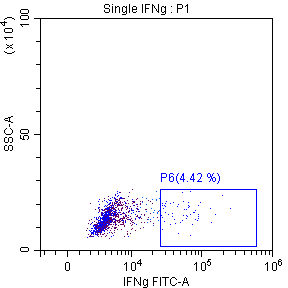 |
| Tbet-PE | 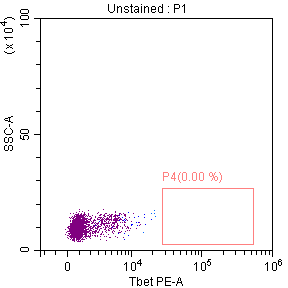 | 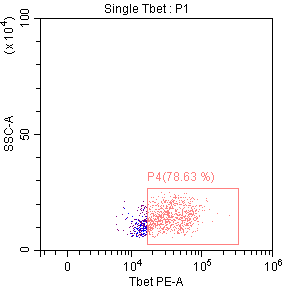 |
| IL-4-FITC | 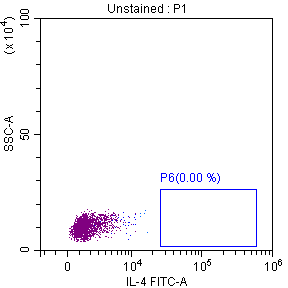 | 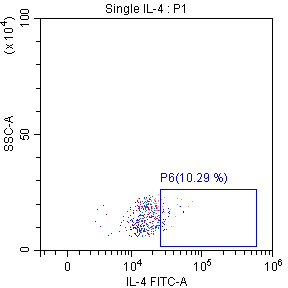 |
| GATA3-PE | 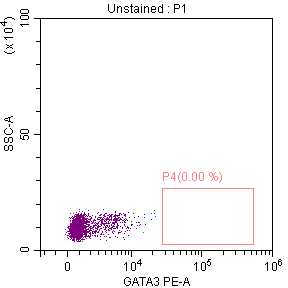 | 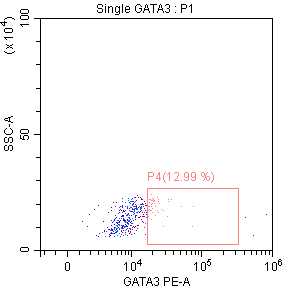 |
| IL-17A-FITC | 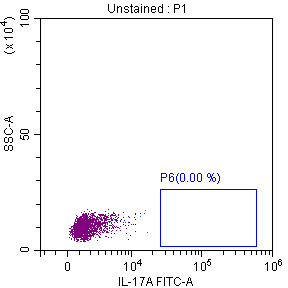 | 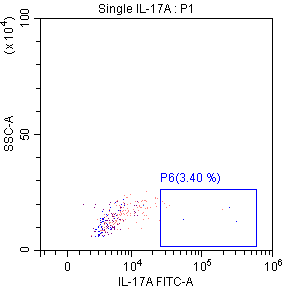 |
| RORC2-PE | 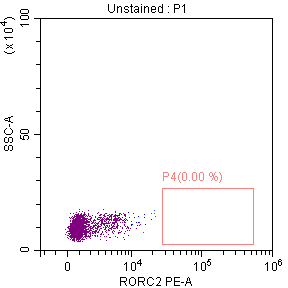 | 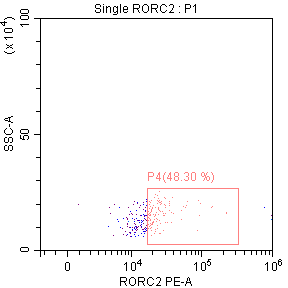 |
| IL-10-PE | 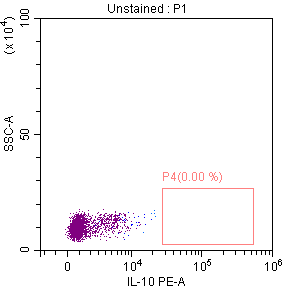 | 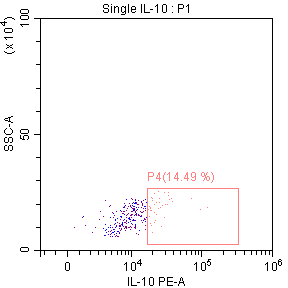 |
| FOXP3-FITC | 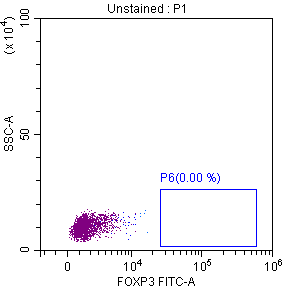 | 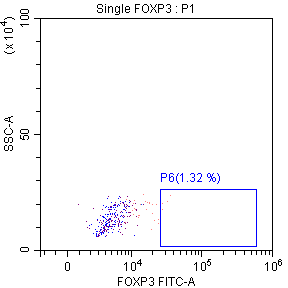 |
| CTLA4-FITC | 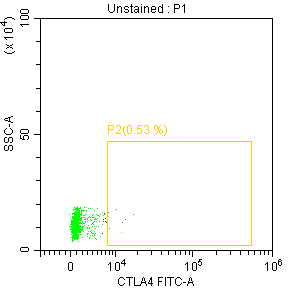 | 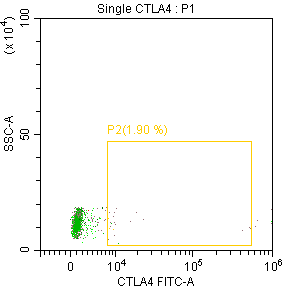 |
| CD80-FITC | 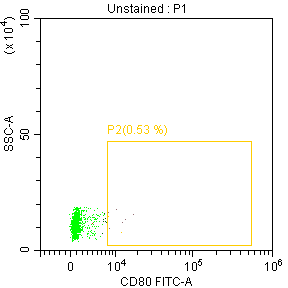 | 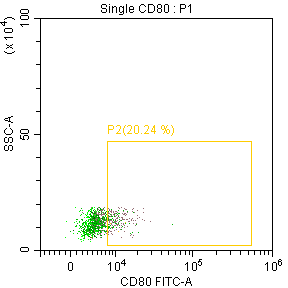 |
| PCNA-PE | 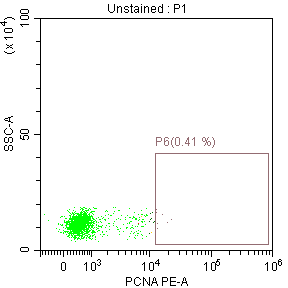 | 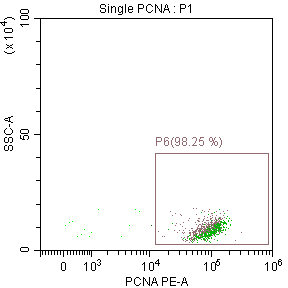 |
| CD86-PE | 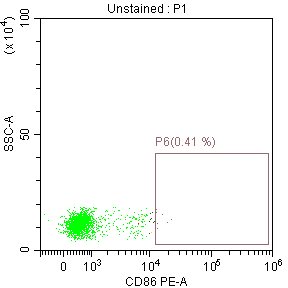 | 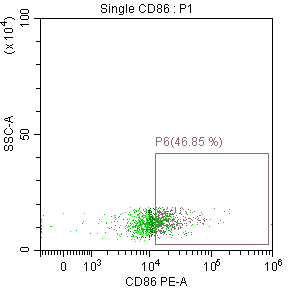 |
| CD69-PE | 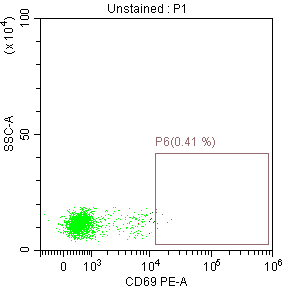 | 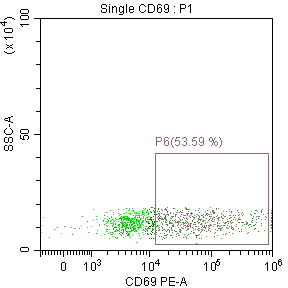 |
| PD1-PE | 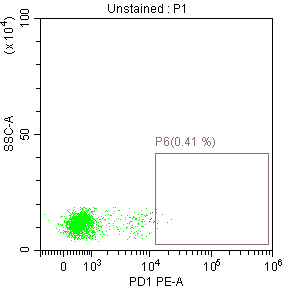 | 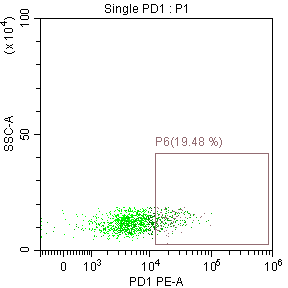 |
