# Supplementary material for: Anti-Th17 and anti-Th2 responses effects of hydro-ethanolic extracts of Aframomum melegueta, Khaya senegalensis and Xylopia aethiopica in hyperreactive onchocerciasis individuals’ peripheral blood mononuclear cells
Source: PLoS Negl Trop Dis. 2022 Apr 25;16(4):e0010341. doi: 10.1371/journal.pntd.0010341 (PMC9071127; doi:10.1371/journal.pntd.0010341)
Supplement: S3 Table — (DOCX) [file pntd.0010341.s006.docx]

**S3_Table:**  Representative plots for the main conditions

| **T Cell Subsets**  **Stimulation** | **Th1** | **Th2** | **Th17** | **Treg** |
| --- | --- | --- | --- | --- |
| **Medium** | 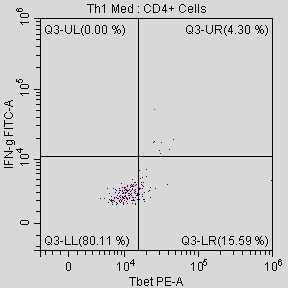 | 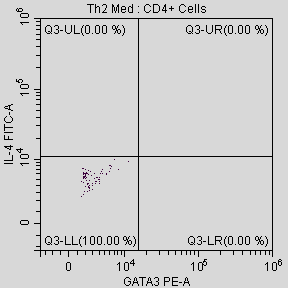 | 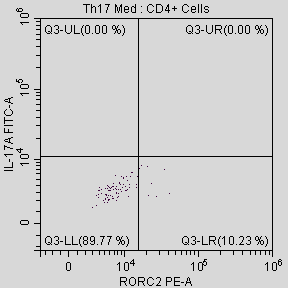 | 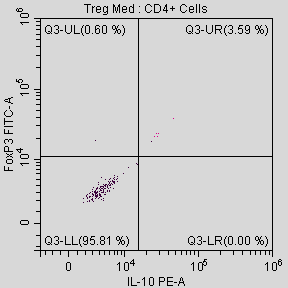 |
| **antiCD3CD28** | 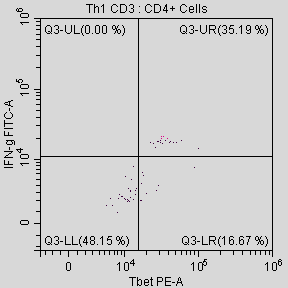 | 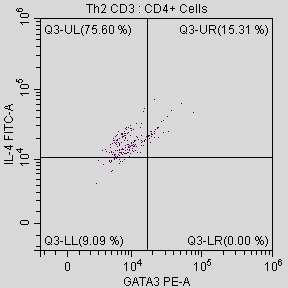 | 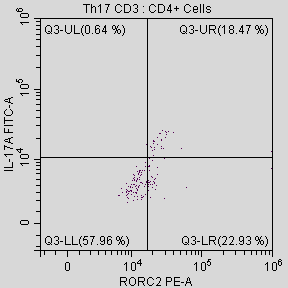 | 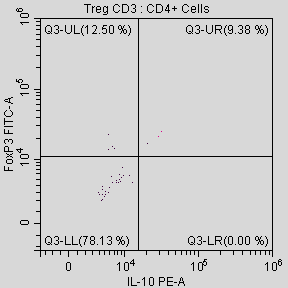 |
| **Ibumol (anti-inflammatory drug )** | 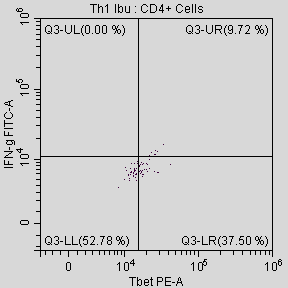 | 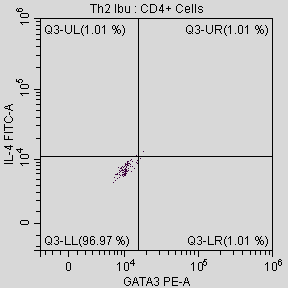 | 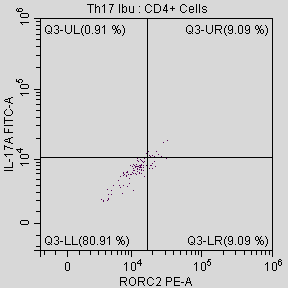 | 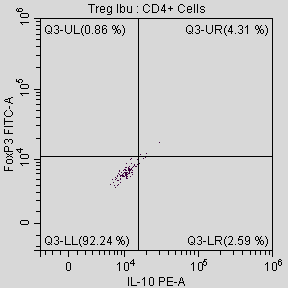 |
| **AM** | 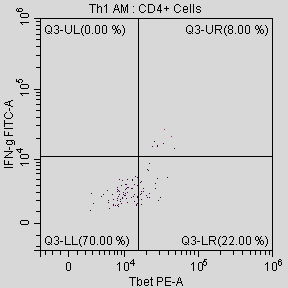 | 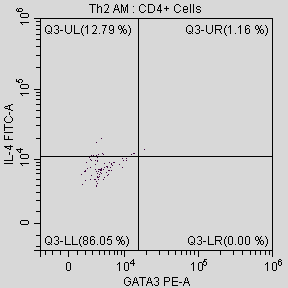 | 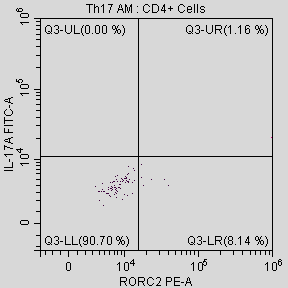 | 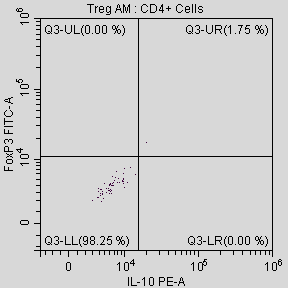 |
| **KS** | 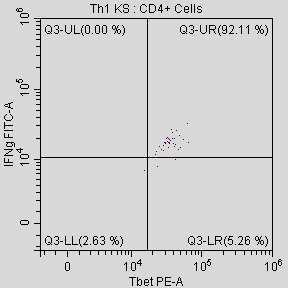 | 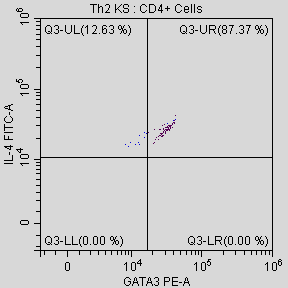 | 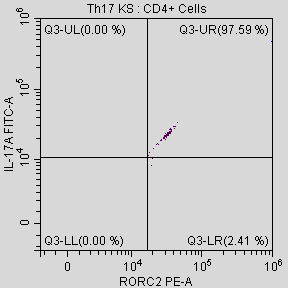 | 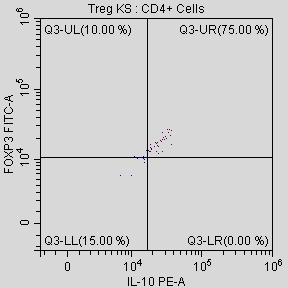 |
| **XA** | 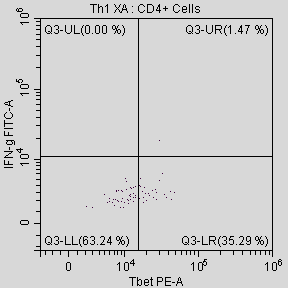 | 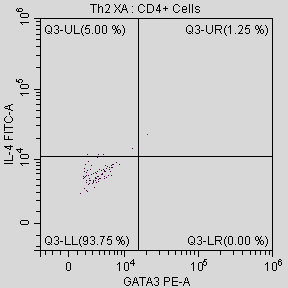 | 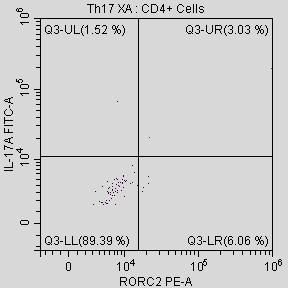 | 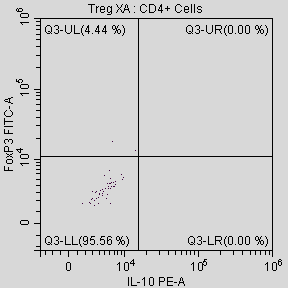 |
